# Crystal structure and Hirshfeld surface analysis of tri­aza­triborinotris[1,3,2]benzodi­aza­borole acetone disolvate

**DOI:** 10.1107/S2056989023009337

**Published:** 2023-10-31

**Authors:** Cole Streeter, Kraig A. Wheeler, Ashley N. Lamm

**Affiliations:** a Department of Chemistry, Biochemistry and Physics, Eastern Washington, University, Cheney, WA 99004, USA; b Department of Chemistry, Whitworth University, Spokane, WA 99251, USA; Universidade de Sâo Paulo, Brazil

**Keywords:** crystal structure, Hirshfeld surface analysis, borazine, benzodi­aza­borole

## Abstract

The title compound, alternatively known as benzodi­aza­borole trimer, C_18_H_15_B_3_N_6_·2C_3_H_6_O, at 100 K crystallizes in the triclinic system, space group *P*  . The structure displays N—H⋯O hydrogen bonding connecting the main mol­ecule with the crystallization solvent. Disorder of the main mol­ecule is observed with occupancy factors refined to 0.8922 (14):0.1078 (14). The packing of the crystal shows a parallel-displaced atom-centered orientation with 3.30 (2) Å between the planes of the rings. In the solid state, the title compound is linked with weak C—H⋯π inter­actions, which is supported by Hirshfeld surface analysis.

## Abstract 2

The title compound, alternatively known as benzodi­aza­borole trimer, C_18_H_15_B_3_N_6_·2C_3_H_6_O, at 100 K crystallizes in the triclinic system, space group *P* 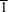 . The structure displays N—H⋯O hydrogen bonding connecting the main mol­ecule with the crystallization solvent.

## 1. Chemical context

Benzodi­aza­borole trimer was discovered over 60 years ago, with the first synthesis reported by Brotherton & Steinberg (1961 ▸). The formation of the borazine ring from three benzodi­aza­boroles is thermodynamically favorable and is found to be the major product (Niedenzu *et al.*, 1962 ▸). There has been research into the physical characteristics, such as IR (Harris & Rudner, 1962 ▸). As a film, the title compound is highly reflective, adherent, hard, and behaves as a narrow-band semiconductor, which is susceptible to modifications by chemical treatment (Maya, 1988 ▸). Additionally, its reactivity and synthesis have been studied by several groups (Kreutzberger & Ferris, 1962 ▸, Ryschkewitsch, 1964 ▸; Trofimenko, 1967 ▸; Dandegaonker & Mane, 1974 ▸; Maras & Kocevar, 2012 ▸). To date, there are no reports on the solid-state structure of the compound. Herein we report on the crystalline structure of the title compound synthesized by the condensation of boron trichloride and *o*-phenyl­enedi­amine and recrystallized in acetone. 
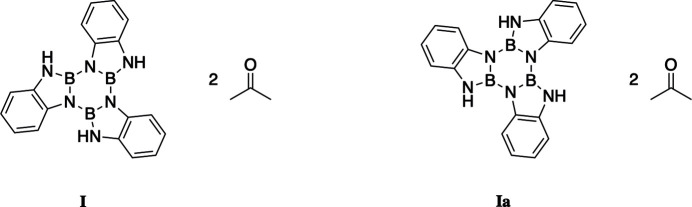


## 2. Structural commentary

**Figure 1:**
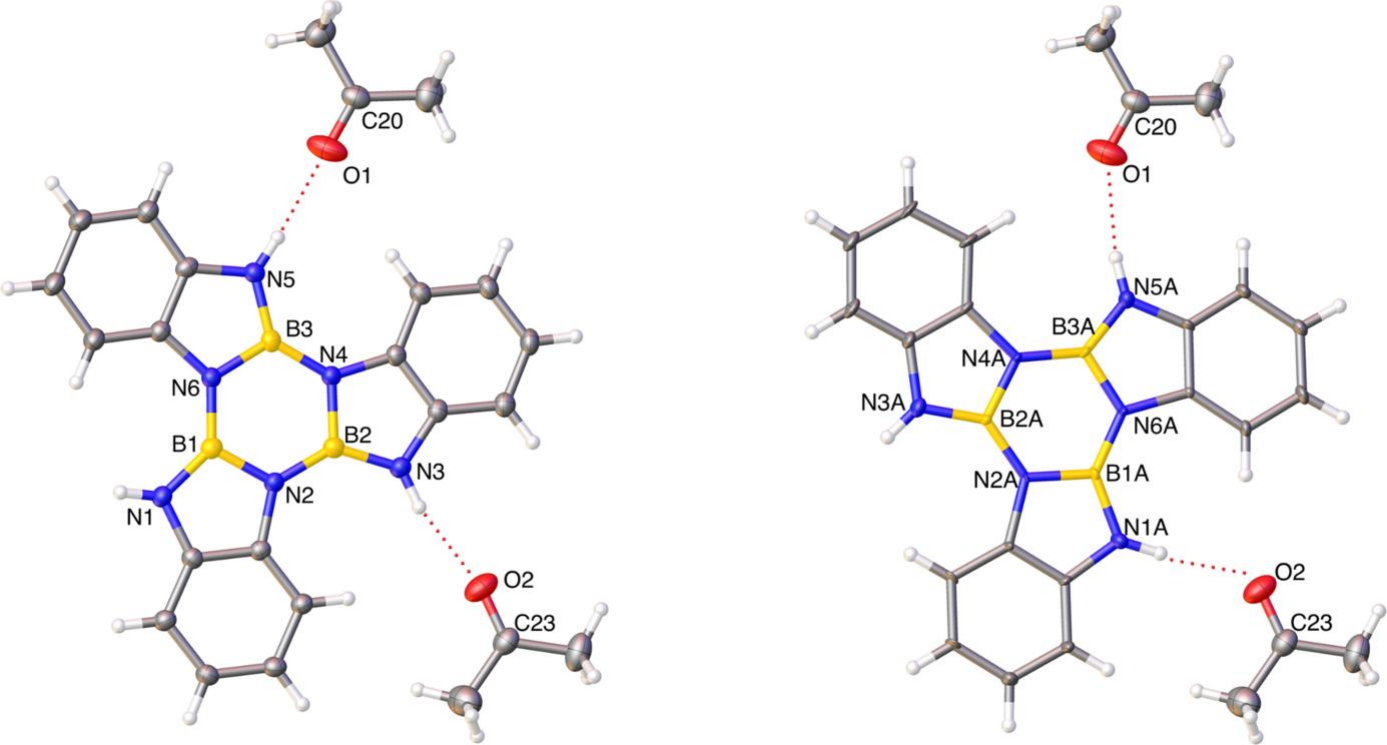
The mol­ecular structure of the disordered title compounds, (**I** and **Ia**), with displacements ellipsoids drawn at the 50% probability level.

The title compound crystallizes with two equivalents of acetone as the crystallization solvent. The structure is disordered, with the acetone mol­ecules remaining stationary while the benzodi­aza­borle is inverted by 180° (Fig. 1 ▸). In each case, the acetone forms hydrogen bonds with a nearby NH group; these bonds range from 2.086 (13) to 2.133 (13) Å. A comparison of between the two mol­ecular orientations, **I** and **Ia**, is given in Table 1 ▸. The C1=O20 double bond of acetone is 1.2110 (15) Å and the O2=C23 double bond is 1.2122 (16) Å. The boron nitro­gen bonds of the borazine ring vary slightly between the disordered structures, **Ia** has slightly shorter bond lengths, see Table 2 ▸. The B—NH bond lengths are similar between the title compounds and vary between 1.4191 (18) and 1.459 (2) Å.

## 3. Supra­molecular features

**Figure 2:**
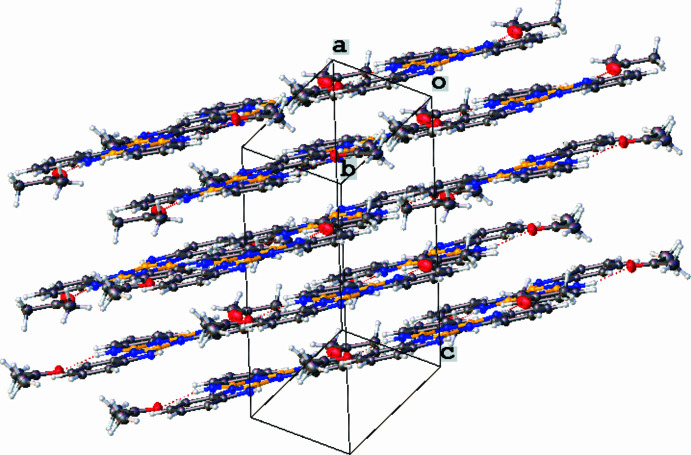
Packing diagram.

**Figure 3:**
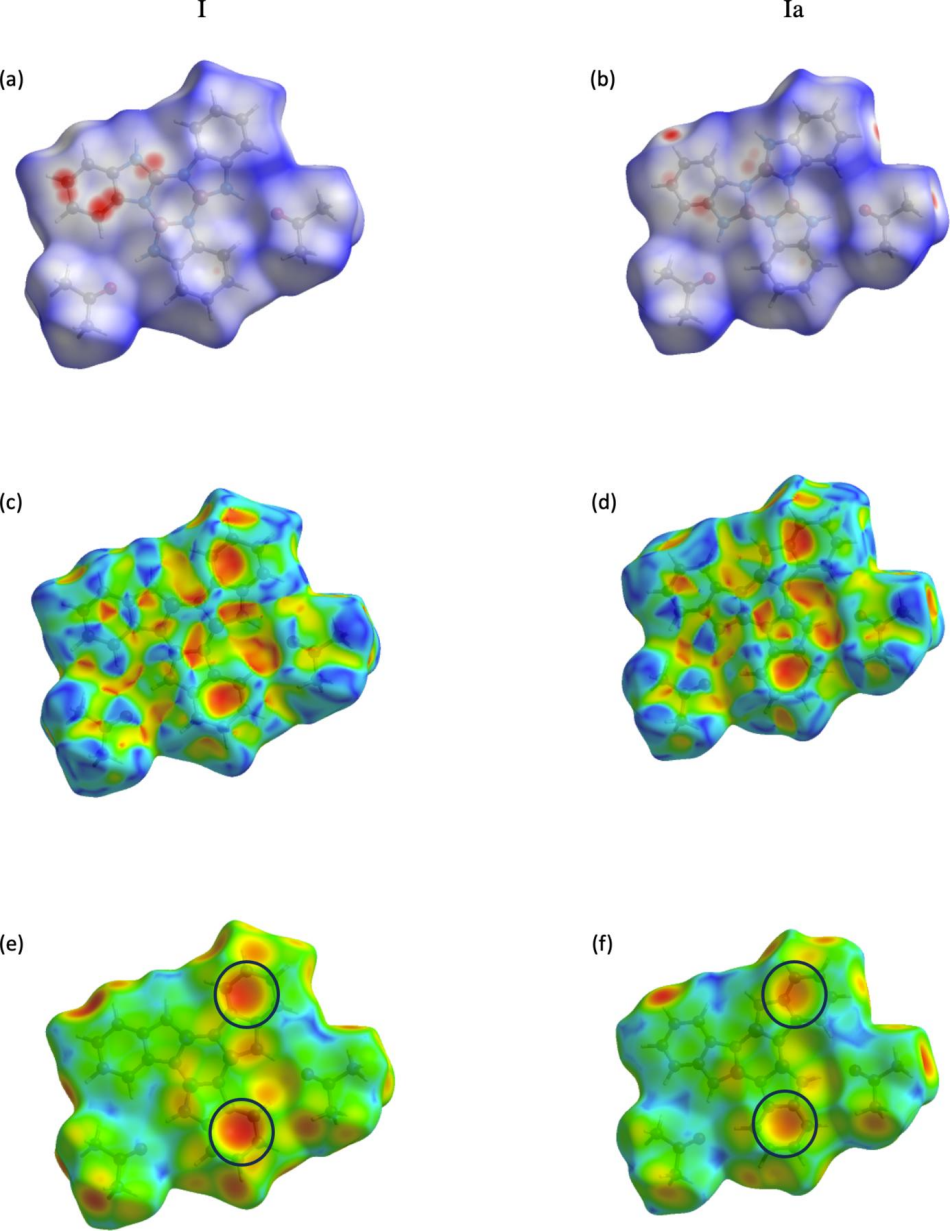
Hirshfeld surface inter­acting mol­ecules mapped over *d*_norm_. Red areas highlight inter­molecular contacts shorter than the sum of the van der Waals radii for **I** (*a*) and **Ia** (*b*), the shape index for **I** (*c*) and **Ia** (*d*), and *d*­_e_ where the circled areas indicate the C—H⋯π inter­actions in **I** (*e*) and **Ia** (*f*).

In the crystal (Fig. 2 ▸), mol­ecules of (I) adopt a parallel-displaced atom-centered orientation. The structure packs with 3.30 (2) Å between adjacent aromatic compounds. The weak inter­molecular inter­actions of **I** and **Ia** were explored by Hirshfeld surface analysis. The properties (*d*_norm_, shape-index and *d*_e_) are mapped over the Hirshfeld surfaces and two-dimensional fingerprint plots of the title compound were generated using *CrystalExplorer* (Version 17.5; Spackman *et al.*, 2021 ▸). The *d*_norm_ values illustrate whether the inter­molecular contact is shorter or longer than the van der Waals radii. Red areas of the Hirshfeld surface indicate negative *d*_norm_ values, which in turn represents contacts closer than the van der Waals radii (Fig. 3 ▸).

**Figure 4:**
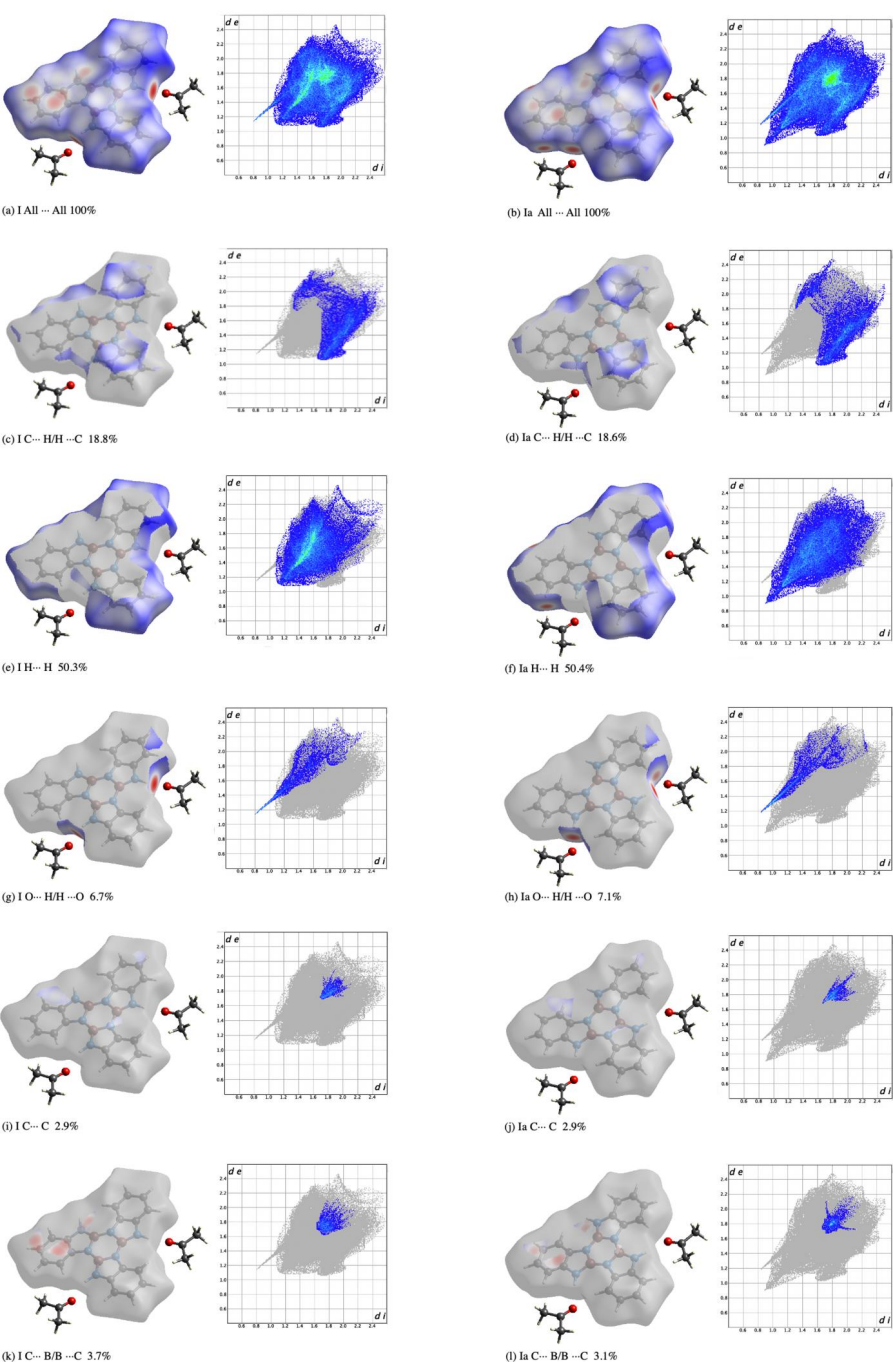
Hirshfeld surfaces and fingerprint plots showing percentage of contacts of all inter­actions for both **I** (*a*) and **Ia** (*b*), C⋯H/ H⋯C inter­actions for **I** (*c*) and **Ia** (*d*), H⋯H inter­actions for **I** (*e*) and **Ia** (*f*), O⋯H/ H⋯O inter­actions for **I** (*g*) and **Ia** (*h*), C⋯C inter­actions for **I** (*i*) and **Ia** (*j*),and C⋯B/ B⋯C inter­actions for **I** (*k*) and **Ia** (*l*)

Contributions by individual elements to inter­molecular inter­actions were analyzed through fingerprint plots generated by *CrystalExplorer* (McKinnon *et al.*, 2004 ▸). The significant inter­actions for each conformer are shown in Fig. 4 ▸(*a*)–(*l*). The dominating inter­actions are H⋯H inter­actions [Fig. 4 ▸(*e*) and (*f*)], followed by C⋯H/ C⋯H inter­actions [Fig. 4 ▸(*c*) and (*d*)], and few C⋯C inter­actions [Fig. 4 ▸(*i*) and (*j*)], which suggests C—H⋯π inter­actions. The hydrogen-bonding inter­actions between O⋯H/O⋯H are very strong [Fig. 4 ▸(*g*) and (*h*)]. Inter­estingly, there are also strong C⋯B/B⋯C inter­actions [Fig. 4 ▸(*k*) and (*l*)]. The negligible contributions from other contacts not included in Fig. 4 ▸ are as follows: N⋯H (**I**, 8.0%; **Ia**, 7.1%), B⋯H (**I**, 3.4%; **Ia** 4.1%),, C⋯O (**I**, 1.0%; **Ia**, 1.1%), N⋯O (**I**, 0.6%; **Ia**, 0.6%), B⋯O (**I**, 0.4%; Ia, 0.3%), C⋯N (**I**, 3.6%; **Ia**, 4.0%), N⋯N (**I**, 0.7%; **Ia** 0.5%), N⋯B (**I**, 0%; **Ia**, 0.4%) with O⋯O, and B⋯B contacts not observed.

## 4. Database survey

A survey of the Cambridge Structural Database (CSD version 2023.2.0; Groom *et al.*, 2016 ▸) showed there are no examples of the benzodi­aza­borole trimer structure and very few examples of similar compounds available in the literature. Crystal structures that contain borazine are usually not fused with five-membered rings except for boraza­truxenes, where the NH groups are replaced with CH_2_ groups. These materials are being explored as stable polycyclic aromatic hydro­carbons for material applications (Limberti *et al.*, 2019 ▸). Additionally, the synthesis of oxaza­borolidine trimers has been studied (Stepanenko *et al.*, 2006 ▸).

## 5. Synthesis and crystallization

A solution of *o*-phenyl­enedi­amine (1.00 g, 9.247 mmol) in 30 mL of toluene with tri­ethyl­amine (3.866 mL, 27.7 mmol) was degassed and boron trichloride (9.247 mL, 9.24 mmol) was slowly added. This mixture was refluxed for 2 h. The cooled solution was filtered through celite and purified by column chromatography with acetone/pentane (1:1) solution. Clear needles were isolated from recrystallization in acetone and were suitable for X-ray diffraction.

## 6. Refinement

Crystal data, data collection and structure refinement details are summarized in Table 3 ▸. All non-hydrogen atoms were located in a series of difference-Fourier electron-density maps and refined using anisotropic displacement parameters. All C—H hydrogen atoms were placed in calculated positions with *U*_iso_(H) = 1.2*U*_eq_ of the connected C atoms (1.5*U*_eq_ for methyl groups). Those H atoms attached to nitro­gen were located in difference-Fourier maps. A RIGU command was applied to the less occupied component and ISOR to N2*A*, N4*A*, and N6*A*. The N—H bonds were restrained with the SADI command. The benzodi­aza­borole mol­ecule was modeled with two-part disorder with occupancy factors refined to 0.8922 (14):0.1078 (14). The minor component was restrained using the SAME and RIGU commands.

## Supplementary Material

Crystal structure: contains datablock(s) I. DOI: 10.1107/S2056989023009337/ex2077sup1.cif

Structure factors: contains datablock(s) I. DOI: 10.1107/S2056989023009337/ex2077Isup2.hkl

Supporting information file. DOI: 10.1107/S2056989023009337/ex2077Isup3.cml

CCDC reference: 2303332

Additional supporting information:  crystallographic information; 3D view; checkCIF report

## Figures and Tables

**Table 1:** Hydrogen-bond geometry (Å, °)

*D*—H⋯*A*	*D*—H	H⋯*A*	*D*⋯*A*	*D*—H⋯*A*
N3—H3⋯O2	0.89 (1)	2.13 (1)	2.9817 (14)	160 (1)
N5—H5⋯O1	0.87 (1)	2.09 (1)	2.9544 (15)	175 (1)
N1*A*—H1*A*⋯O2	0.88 (2)	2.27 (4)	3.133 (8)	165 (11)
N5*A*—H5*AA*⋯O1	0.88 (2)	2.19 (5)	3.026 (8)	158 (11)

**Table 2:** Comparison of borazine bond lengths (Å) in **I** and **Ia**

**I**		**Ia**	
N2—B1	1.459 (2)	N2*A*—B1*A*	1.433 (12)
N2—B2	1.429 (2)	N2*A*—B2*A*	1.433 (12)
N4—B2	1.459 (2)	N4*A*—B2*A*	1.433 (13)
N4—B3	1.433 (2)	N4*A*—B3*A*	1.429 (12)
N6—B1	1.428 (2)	N6*A*—B1*A*	1.409 (12)
N6—B3	1.459 (2)	N6*A*—B3*A*	1.435 (12)

**Table 3:** Experimental details

Crystal data	
Chemical formula	C_18_H_15_B_3_N_6_·2C_3_H_6_O
*M*_r_	463.94
Crystal system, space group	Triclinic, *P* 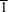
Temperature (K)	100
*a*, *b*, *c* (Å)	5.8357 (4), 13.6362 (9), 15.4653 (10)
α, β, γ (°)	97.595 (3), 96.957 (3), 99.771 (3)
*V* (Å^3^)	1189.13 (14)
*Z*	2
Radiation type	Cu *K*α
μ (mm^−1^)	0.66
Crystal size (mm)	0.41 × 0.13 × 0.13

Data collection	
Diffractometer	Bruker D8 Venture
Absorption correction	Multi-scan (*SADABS*; Krause *et al.*, 2015 ▸)
*T*_min_, *T*_max_	0.699, 0.754
No. of measured, independent and observed [*I* > 2σ(*I*)] reflections	47276, 4853, 4226
*R*_int_	0.040
(sin θ/λ)_max_ (Å^−1^)	0.625

Refinement	
*R*[*F* ^2^ > 2σ(*F* ^2^)], *wR*(*F* ^2^), *S*	0.039, 0.095, 1.08
No. of reflections	4853
No. of parameters	579
No. of restraints	369
H-atom treatment	H atoms treated by a mixture of independent and constrained refinement
Δρ_max_, Δρ_min_ (e Å^−3^)	0.22, −0.23

Computer programs: *APEX4* (Bruker, 2022 ▸), *SAINT-Plus* (Bruker, 2020 ▸), *SHELXT2018/2* (Sheldrick, 2015*a* ▸), *SHELXL209/1* (Sheldrick, 2015*b* ▸), and *OLEX2* (Dolomanov *et al.*, 2009 ▸).

## supplementary crystallographic information

### Crystal data

**Table:**

C_18_H_15_B_3_N_6_·2C_3_H_6_O	*Z* = 2
*M**_r_* = 463.94	*F*(000) = 488
Triclinic, *P*1	*D*_x_ = 1.296 Mg m^−^^3^
*a* = 5.8357 (4) Å	Cu *K*α radiation, λ = 1.54178 Å
*b* = 13.6362 (9) Å	Cell parameters from 9824 reflections
*c* = 15.4653 (10) Å	θ = 3.3–74.3°
α = 97.595 (3)°	µ = 0.66 mm^−^^1^
β = 96.957 (3)°	*T* = 100 K
γ = 99.771 (3)°	Rod, colourless
*V* = 1189.13 (14) Å^3^	0.41 × 0.13 × 0.13 mm

### Data collection

**Table:**

Bruker D8 Venture diffractometer	4853 independent reflections
Radiation source: microfocus sealed X-ray tube, Incoatec IµS 3.0	4226 reflections with *I* > 2σ(*I*)
Multilayer mirror monochromator	*R*_int_ = 0.040
Detector resolution: 7.9 pixels mm^-1^	θ_max_ = 74.6°, θ_min_ = 2.9°
ω and φ scans	*h* = −7→7
Absorption correction: multi-scan (SADABS; Krause *et al.*, 2015)	*k* = −17→17
*T*_min_ = 0.699, *T*_max_ = 0.754	*l* = −19→19
47276 measured reflections	

### Refinement

**Table:**

Refinement on *F*^2^	Primary atom site location: dual
Least-squares matrix: full	Hydrogen site location: mixed
*R*[*F*^2^ > 2σ(*F*^2^)] = 0.039	H atoms treated by a mixture of independent and constrained refinement
*wR*(*F*^2^) = 0.095	*w* = 1/[σ^2^(*F*_o_^2^) + (0.0377*P*)^2^ + 0.3978*P*] where *P* = (*F*_o_^2^ + 2*F*_c_^2^)/3
*S* = 1.08	(Δ/σ)_max_ < 0.001
4853 reflections	Δρ_max_ = 0.22 e Å^−^^3^
579 parameters	Δρ_min_ = −0.23 e Å^−^^3^
369 restraints	

### Special details

**Table:**

Geometry. All esds (except the esd in the dihedral angle between two l.s. planes) are estimated using the full covariance matrix. The cell esds are taken into account individually in the estimation of esds in distances, angles and torsion angles; correlations between esds in cell parameters are only used when they are defined by crystal symmetry. An approximate (isotropic) treatment of cell esds is used for estimating esds involving l.s. planes.
Refinement. All nonhydrogen atoms were located in a series of difference Fourier electron density maps and refined using anisotropic displacement parameters. All C-H hydrogen atoms were placed in calculated positions with Uiso = 1.2xUeqiv of the connected C atoms 1.5xUeqiv for methyl groups). Those H atoms attached to nitrogen were located in Fourier diff maps. A RIGU command was applied to the minor component and ISOR to N2A, N4A, and N6A. The N-H bonds were restrained with the SADI command. The benzodiazaborole molecule was modeled with two-part disorder with occupancy factors refined to 89:11. The minor restrained using the SAME and RIGU commands. N2A, N4A, N6A were also restrained with an ISOR comand. The locations NH hydrogen atoms of the minor componenent were were fixed using AFIX 43 commands.

### Fractional atomic coordinates and isotropic or equivalent isotropic displacement parameters (Å^2^)

**Table:**

	*x*	*y*	*z*	*U*_iso_*/*U*_eq_	Occ. (<1)
O1	1.06089 (19)	0.80696 (9)	0.44502 (7)	0.0509 (3)	
C19	1.4443 (2)	0.84484 (11)	0.52511 (9)	0.0394 (3)	
H19A	1.465884	0.900588	0.491055	0.059*	
H19B	1.566486	0.804633	0.517058	0.059*	
H19C	1.455742	0.871885	0.587746	0.059*	
C20	1.2081 (2)	0.78000 (10)	0.49384 (8)	0.0292 (3)	
C21	1.1621 (3)	0.68044 (11)	0.52471 (10)	0.0414 (3)	
H21A	0.992312	0.654847	0.516480	0.062*	
H21B	1.225795	0.688200	0.587362	0.062*	
H21C	1.238069	0.632812	0.490587	0.062*	
O2	−0.42413 (17)	0.45535 (8)	0.15098 (7)	0.0438 (3)	
C22	−0.7168 (3)	0.31164 (10)	0.15045 (10)	0.0444 (4)	
H22A	−0.746614	0.260253	0.097682	0.067*	
H22B	−0.863479	0.314031	0.174805	0.067*	
H22C	−0.600541	0.294781	0.194562	0.067*	
C23	−0.6251 (2)	0.41159 (9)	0.12665 (8)	0.0307 (3)	
C24	−0.7921 (3)	0.45521 (12)	0.06874 (11)	0.0502 (4)	
H24A	−0.911786	0.476703	0.102329	0.075*	
H24B	−0.868343	0.404166	0.018179	0.075*	
H24C	−0.705855	0.513364	0.047717	0.075*	
N1	0.05231 (18)	0.94365 (8)	0.12177 (7)	0.0213 (2)	0.8922 (14)
H1	0.087 (3)	1.0065 (10)	0.1141 (10)	0.026*	0.8922 (14)
N2	0.0215 (2)	0.79057 (11)	0.17389 (8)	0.0199 (3)	0.8922 (14)
N3	−0.01420 (19)	0.61709 (8)	0.23107 (7)	0.0223 (2)	0.8922 (14)
H3	−0.155 (2)	0.5811 (11)	0.2091 (10)	0.027*	0.8922 (14)
N4	0.3305 (3)	0.73686 (10)	0.27224 (7)	0.0199 (3)	0.8922 (14)
N5	0.70842 (18)	0.87612 (8)	0.32562 (7)	0.0217 (2)	0.8922 (14)
H5	0.808 (2)	0.8516 (11)	0.3595 (9)	0.026*	0.8922 (14)
N6	0.3973 (2)	0.90929 (9)	0.23233 (8)	0.0194 (2)	0.8922 (14)
C1	−0.1652 (3)	0.88371 (13)	0.08577 (11)	0.0198 (3)	0.8922 (14)
C2	−0.3450 (4)	0.90431 (14)	0.02761 (13)	0.0224 (4)	0.8922 (14)
H2	−0.329678	0.966365	0.005266	0.027*	0.8922 (14)
C3	−0.5501 (3)	0.83056 (16)	0.00289 (13)	0.0229 (4)	0.8922 (14)
H3A	−0.676721	0.843184	−0.036131	0.027*	0.8922 (14)
C4	−0.5705 (3)	0.73916 (13)	0.03476 (11)	0.0229 (4)	0.8922 (14)
H4	−0.710825	0.690246	0.017142	0.027*	0.8922 (14)
C5	−0.3876 (3)	0.71828 (12)	0.09227 (10)	0.0212 (3)	0.8922 (14)
H5A	−0.401702	0.655786	0.113858	0.025*	0.8922 (14)
C6	−0.1858 (3)	0.79089 (14)	0.11696 (8)	0.0191 (3)	0.8922 (14)
C7	0.1469 (3)	0.57793 (15)	0.28473 (8)	0.0208 (3)	0.8922 (14)
C8	0.1229 (3)	0.48537 (15)	0.31331 (13)	0.0244 (4)	0.8922 (14)
H8	−0.020650	0.438014	0.298263	0.029*	0.8922 (14)
C9	0.3158 (4)	0.46330 (17)	0.36499 (18)	0.0260 (5)	0.8922 (14)
H9	0.303614	0.400122	0.385324	0.031*	0.8922 (14)
C10	0.5261 (3)	0.53338 (15)	0.38692 (12)	0.0251 (4)	0.8922 (14)
H10	0.656080	0.516669	0.421237	0.030*	0.8922 (14)
C11	0.5489 (3)	0.62773 (11)	0.35930 (10)	0.0227 (3)	0.8922 (14)
H11	0.691815	0.675431	0.374747	0.027*	0.8922 (14)
C12	0.3572 (3)	0.64948 (10)	0.30890 (10)	0.0199 (3)	0.8922 (14)
C13	0.7668 (4)	0.97371 (11)	0.30686 (11)	0.0215 (3)	0.8922 (14)
C14	0.9721 (4)	1.04497 (16)	0.33531 (14)	0.0244 (4)	0.8922 (14)
H14	1.097907	1.031074	0.373927	0.029*	0.8922 (14)
C15	0.9867 (4)	1.13764 (18)	0.30511 (17)	0.0262 (5)	0.8922 (14)
H15	1.125364	1.187461	0.322980	0.031*	0.8922 (14)
C16	0.8000 (3)	1.15820 (13)	0.24893 (11)	0.0266 (4)	0.8922 (14)
H16	0.813959	1.221762	0.229232	0.032*	0.8922 (14)
C17	0.5933 (3)	1.08661 (14)	0.22134 (9)	0.0240 (3)	0.8922 (14)
H17	0.466136	1.100974	0.183733	0.029*	0.8922 (14)
C18	0.5792 (2)	0.99461 (14)	0.25019 (10)	0.0200 (3)	0.8922 (14)
B1	0.1734 (3)	0.88827 (13)	0.17792 (10)	0.0203 (3)	0.8922 (14)
B2	0.0951 (3)	0.71571 (12)	0.22164 (12)	0.0206 (3)	0.8922 (14)
B3	0.4808 (3)	0.83321 (14)	0.27939 (10)	0.0202 (3)	0.8922 (14)
N1A	−0.1999 (13)	0.6816 (6)	0.1539 (5)	0.0194 (17)	0.1078 (14)
H1A	−0.282 (18)	0.623 (4)	0.158 (8)	0.023*	0.1078 (14)
N2A	0.0651 (16)	0.8336 (8)	0.1694 (6)	0.0115 (18)	0.1078 (14)
N3A	0.3577 (13)	1.0072 (6)	0.1865 (5)	0.0199 (17)	0.1078 (14)
H3AA	0.282922	1.041925	0.152282	0.024*	0.1078 (14)
N4A	0.4546 (15)	0.8803 (7)	0.2592 (6)	0.0101 (16)	0.1078 (14)
N5A	0.5854 (14)	0.7321 (6)	0.3329 (6)	0.0202 (18)	0.1078 (14)
H5AA	0.722 (10)	0.736 (9)	0.366 (7)	0.024*	0.1078 (14)
N6A	0.2152 (19)	0.7135 (6)	0.2538 (6)	0.0106 (17)	0.1078 (14)
C1A	−0.290 (2)	0.7472 (10)	0.1031 (7)	0.014 (2)	0.1078 (14)
C2A	−0.506 (3)	0.7254 (10)	0.0533 (11)	0.022 (3)	0.1078 (14)
H2A	−0.609929	0.663375	0.052168	0.026*	0.1078 (14)
C3A	−0.568 (3)	0.7960 (12)	0.0050 (13)	0.022 (3)	0.1078 (14)
H3AB	−0.715553	0.783290	−0.031800	0.026*	0.1078 (14)
C4A	−0.417 (3)	0.8847 (12)	0.0099 (11)	0.017 (3)	0.1078 (14)
H4A	−0.463478	0.933462	−0.023647	0.021*	0.1078 (14)
C5A	−0.201 (2)	0.9069 (10)	0.0609 (9)	0.014 (2)	0.1078 (14)
H5AB	−0.096661	0.968971	0.062466	0.017*	0.1078 (14)
C6A	−0.1419 (18)	0.8371 (10)	0.1089 (7)	0.013 (2)	0.1078 (14)
C7A	0.5800 (17)	1.0397 (11)	0.2354 (8)	0.018 (2)	0.1078 (14)
C8A	0.720 (3)	1.1301 (10)	0.2390 (10)	0.025 (3)	0.1078 (14)
H8A	0.670904	1.178949	0.206266	0.030*	0.1078 (14)
C9A	0.935 (3)	1.1498 (14)	0.2911 (15)	0.020 (3)	0.1078 (14)
H9A	1.036852	1.213402	0.296635	0.025*	0.1078 (14)
C10A	1.000 (3)	1.0759 (13)	0.3346 (15)	0.025 (4)	0.1078 (14)
H10A	1.151947	1.089307	0.368572	0.030*	0.1078 (14)
C11A	0.860 (2)	0.9829 (10)	0.3327 (9)	0.020 (3)	0.1078 (14)
H11A	0.908625	0.933801	0.365401	0.024*	0.1078 (14)
C12A	0.647 (2)	0.9667 (8)	0.2801 (8)	0.012 (2)	0.1078 (14)
C13A	0.462 (2)	0.6377 (8)	0.3348 (8)	0.015 (2)	0.1078 (14)
C14A	0.542 (2)	0.5652 (11)	0.3771 (11)	0.018 (3)	0.1078 (14)
H14A	0.697182	0.574882	0.407814	0.022*	0.1078 (14)
C15A	0.382 (3)	0.4774 (15)	0.3719 (16)	0.019 (3)	0.1078 (14)
H15A	0.429636	0.425514	0.400910	0.022*	0.1078 (14)
C16A	0.156 (3)	0.4599 (12)	0.3273 (13)	0.020 (3)	0.1078 (14)
H16A	0.053894	0.397267	0.325790	0.023*	0.1078 (14)
C17A	0.077 (2)	0.5340 (10)	0.2846 (8)	0.016 (2)	0.1078 (14)
H17A	−0.077468	0.524471	0.253539	0.019*	0.1078 (14)
C18A	0.236 (2)	0.6213 (8)	0.2900 (6)	0.0082 (18)	0.1078 (14)
B1A	0.0310 (19)	0.7366 (9)	0.1970 (8)	0.011 (2)	0.1078 (14)
B2A	0.273 (2)	0.9081 (8)	0.2018 (8)	0.010 (2)	0.1078 (14)
B3A	0.434 (2)	0.7822 (9)	0.2840 (7)	0.012 (2)	0.1078 (14)

### Atomic displacement parameters (Å^2^)

**Table:**

	*U*^11^	*U*^22^	*U*^33^	*U*^12^	*U*^13^	*U*^23^
O1	0.0489 (6)	0.0642 (7)	0.0388 (6)	0.0269 (5)	−0.0118 (5)	0.0005 (5)
C19	0.0371 (7)	0.0432 (8)	0.0344 (7)	0.0001 (6)	0.0072 (6)	0.0006 (6)
C20	0.0295 (6)	0.0356 (7)	0.0227 (6)	0.0107 (5)	0.0019 (5)	0.0010 (5)
C21	0.0511 (9)	0.0364 (8)	0.0373 (7)	0.0044 (6)	0.0156 (6)	0.0045 (6)
O2	0.0341 (5)	0.0417 (6)	0.0466 (6)	−0.0080 (4)	0.0031 (4)	−0.0040 (5)
C22	0.0566 (9)	0.0283 (7)	0.0492 (9)	0.0015 (6)	0.0233 (7)	0.0035 (6)
C23	0.0324 (7)	0.0251 (6)	0.0322 (6)	0.0013 (5)	0.0069 (5)	−0.0012 (5)
C24	0.0539 (9)	0.0412 (8)	0.0520 (9)	0.0131 (7)	−0.0053 (7)	0.0006 (7)
N1	0.0204 (5)	0.0182 (5)	0.0245 (5)	0.0017 (4)	0.0015 (4)	0.0043 (4)
N2	0.0173 (6)	0.0192 (7)	0.0226 (6)	0.0017 (5)	0.0019 (4)	0.0048 (6)
N3	0.0193 (5)	0.0217 (5)	0.0252 (5)	0.0017 (4)	0.0012 (4)	0.0053 (4)
N4	0.0181 (7)	0.0201 (6)	0.0214 (6)	0.0023 (5)	0.0016 (5)	0.0060 (5)
N5	0.0202 (5)	0.0221 (5)	0.0223 (5)	0.0028 (4)	0.0002 (4)	0.0059 (4)
N6	0.0192 (6)	0.0189 (6)	0.0199 (6)	0.0024 (5)	0.0020 (5)	0.0046 (5)
C1	0.0204 (7)	0.0201 (9)	0.0195 (8)	0.0055 (6)	0.0033 (6)	0.0026 (6)
C2	0.0201 (10)	0.0215 (8)	0.0243 (9)	0.0029 (7)	0.0014 (8)	0.0020 (7)
C3	0.0217 (8)	0.0249 (10)	0.0222 (7)	0.0059 (8)	0.0005 (6)	0.0044 (8)
C4	0.0214 (8)	0.0254 (9)	0.0202 (8)	0.0029 (6)	0.0002 (6)	0.0021 (6)
C5	0.0205 (8)	0.0208 (7)	0.0211 (7)	0.0023 (6)	0.0015 (6)	0.0019 (6)
C6	0.0182 (7)	0.0195 (8)	0.0194 (6)	0.0035 (6)	0.0032 (5)	0.0018 (6)
C7	0.0237 (8)	0.0186 (8)	0.0204 (7)	0.0028 (7)	0.0057 (6)	0.0030 (7)
C8	0.0270 (8)	0.0225 (10)	0.0244 (10)	0.0037 (7)	0.0053 (7)	0.0055 (7)
C9	0.0331 (13)	0.0193 (9)	0.0262 (10)	0.0025 (8)	0.0060 (10)	0.0074 (8)
C10	0.0303 (9)	0.0230 (10)	0.0238 (8)	0.0078 (8)	0.0024 (6)	0.0078 (7)
C11	0.0245 (7)	0.0227 (8)	0.0216 (7)	0.0056 (6)	0.0029 (6)	0.0053 (6)
C12	0.0209 (8)	0.0202 (7)	0.0190 (7)	0.0038 (6)	0.0039 (6)	0.0042 (5)
C13	0.0191 (9)	0.0231 (7)	0.0203 (8)	0.0005 (7)	0.0031 (7)	0.0006 (6)
C14	0.0232 (9)	0.0224 (10)	0.0262 (8)	0.0010 (8)	0.0030 (7)	0.0038 (9)
C15	0.0234 (10)	0.0245 (10)	0.0272 (11)	−0.0020 (7)	0.0003 (7)	0.0029 (7)
C16	0.0271 (9)	0.0217 (8)	0.0276 (8)	−0.0034 (7)	0.0022 (7)	0.0036 (6)
C17	0.0250 (8)	0.0213 (8)	0.0235 (7)	−0.0003 (6)	0.0013 (5)	0.0035 (6)
C18	0.0205 (7)	0.0196 (8)	0.0194 (7)	0.0022 (6)	0.0035 (5)	0.0024 (6)
B1	0.0207 (8)	0.0208 (8)	0.0192 (7)	0.0031 (7)	0.0041 (6)	0.0017 (6)
B2	0.0195 (9)	0.0227 (8)	0.0200 (8)	0.0040 (6)	0.0038 (7)	0.0037 (6)
B3	0.0225 (7)	0.0204 (9)	0.0190 (7)	0.0049 (7)	0.0056 (5)	0.0049 (7)
N1A	0.018 (3)	0.016 (3)	0.022 (3)	0.001 (2)	−0.006 (2)	0.007 (3)
N2A	0.011 (3)	0.007 (3)	0.015 (3)	−0.001 (2)	−0.007 (2)	0.008 (2)
N3A	0.019 (3)	0.014 (3)	0.024 (4)	−0.002 (2)	0.000 (3)	0.006 (2)
N4A	0.007 (3)	0.008 (3)	0.013 (3)	−0.0032 (19)	−0.004 (2)	0.005 (2)
N5A	0.016 (3)	0.018 (3)	0.026 (4)	0.001 (2)	−0.002 (2)	0.010 (3)
N6A	0.008 (3)	0.007 (3)	0.015 (3)	−0.002 (2)	−0.005 (2)	0.005 (2)
C1A	0.010 (3)	0.010 (4)	0.017 (4)	−0.004 (2)	−0.007 (3)	0.005 (3)
C2A	0.016 (4)	0.014 (4)	0.029 (6)	−0.006 (3)	−0.014 (3)	0.011 (4)
C3A	0.014 (4)	0.020 (5)	0.028 (6)	−0.002 (4)	−0.008 (3)	0.012 (5)
C4A	0.009 (4)	0.016 (5)	0.027 (6)	0.004 (3)	−0.007 (3)	0.012 (4)
C5A	0.009 (4)	0.014 (4)	0.020 (5)	0.005 (3)	−0.003 (3)	0.010 (3)
C6A	0.010 (3)	0.007 (4)	0.020 (4)	−0.002 (2)	−0.009 (3)	0.007 (3)
C7A	0.015 (3)	0.011 (3)	0.024 (5)	−0.004 (3)	−0.003 (3)	0.007 (3)
C8A	0.020 (4)	0.014 (4)	0.034 (6)	−0.009 (3)	−0.005 (4)	0.007 (3)
C9A	0.019 (5)	0.014 (5)	0.025 (6)	−0.008 (3)	0.000 (4)	0.004 (4)
C10A	0.015 (4)	0.017 (5)	0.036 (7)	−0.012 (3)	−0.005 (4)	0.008 (5)
C11A	0.010 (4)	0.018 (4)	0.026 (5)	−0.008 (3)	−0.005 (3)	0.005 (4)
C12A	0.008 (3)	0.010 (3)	0.016 (4)	−0.004 (2)	−0.001 (3)	0.003 (3)
C13A	0.009 (4)	0.016 (3)	0.021 (5)	0.001 (2)	−0.005 (3)	0.011 (3)
C14A	0.016 (4)	0.015 (4)	0.022 (5)	0.001 (3)	−0.007 (3)	0.010 (4)
C15A	0.018 (5)	0.016 (4)	0.022 (6)	0.003 (3)	−0.004 (4)	0.010 (4)
C16A	0.021 (5)	0.014 (4)	0.024 (6)	0.003 (3)	−0.003 (4)	0.012 (4)
C17A	0.016 (4)	0.011 (4)	0.020 (5)	0.000 (3)	−0.001 (3)	0.010 (3)
C18A	0.008 (3)	0.008 (3)	0.009 (4)	0.001 (2)	−0.001 (3)	0.004 (3)
B1A	0.011 (3)	0.008 (3)	0.013 (4)	0.001 (2)	−0.003 (3)	0.006 (3)
B2A	0.008 (3)	0.008 (3)	0.013 (4)	−0.001 (2)	−0.003 (3)	0.004 (2)
B3A	0.009 (3)	0.009 (3)	0.016 (4)	−0.003 (2)	−0.004 (3)	0.005 (3)

### Geometric parameters (Å, º)

**Table:**

O1—C20	1.2110 (15)	C13—C18	1.410 (2)
C19—H19A	0.9800	C14—H14	0.9500
C19—H19B	0.9800	C14—C15	1.397 (2)
C19—H19C	0.9800	C15—H15	0.9500
C19—C20	1.4927 (18)	C15—C16	1.400 (2)
C20—C21	1.4906 (19)	C16—H16	0.9500
C21—H21A	0.9800	C16—C17	1.398 (2)
C21—H21B	0.9800	C17—H17	0.9500
C21—H21C	0.9800	C17—C18	1.379 (2)
O2—C23	1.2122 (16)	N1A—H1A	0.880 (18)
C22—H22A	0.9800	N1A—C1A	1.391 (11)
C22—H22B	0.9800	N1A—B1A	1.460 (12)
C22—H22C	0.9800	N2A—C6A	1.447 (11)
C22—C23	1.4873 (18)	N2A—B1A	1.433 (12)
C23—C24	1.494 (2)	N2A—B2A	1.433 (12)
C24—H24A	0.9800	N3A—H3AA	0.8800
C24—H24B	0.9800	N3A—C7A	1.389 (11)
C24—H24C	0.9800	N3A—B2A	1.419 (11)
N1—H1	0.874 (12)	N4A—C12A	1.454 (11)
N1—C1	1.3948 (18)	N4A—B2A	1.433 (13)
N1—B1	1.4247 (19)	N4A—B3A	1.429 (12)
N2—C6	1.4082 (17)	N5A—H5AA	0.883 (18)
N2—B1	1.459 (2)	N5A—C13A	1.370 (11)
N2—B2	1.429 (2)	N5A—B3A	1.414 (11)
N3—H3	0.886 (12)	N6A—C18A	1.459 (11)
N3—C7	1.3985 (17)	N6A—B1A	1.409 (12)
N3—B2	1.4212 (19)	N6A—B3A	1.435 (12)
N4—C12	1.4091 (18)	C1A—C2A	1.362 (13)
N4—B2	1.459 (2)	C1A—C6A	1.359 (13)
N4—B3	1.433 (2)	C2A—H2A	0.9500
N5—H5	0.871 (12)	C2A—C3A	1.363 (14)
N5—C13	1.3941 (17)	C3A—H3AB	0.9500
N5—B3	1.4191 (18)	C3A—C4A	1.356 (14)
N6—C18	1.4077 (18)	C4A—H4A	0.9500
N6—B1	1.428 (2)	C4A—C5A	1.369 (13)
N6—B3	1.459 (2)	C5A—H5AB	0.9500
C1—C2	1.388 (2)	C5A—C6A	1.346 (12)
C1—C6	1.405 (2)	C7A—C8A	1.349 (13)
C2—H2	0.9500	C7A—C12A	1.367 (13)
C2—C3	1.403 (2)	C8A—H8A	0.9500
C3—H3A	0.9500	C8A—C9A	1.371 (15)
C3—C4	1.392 (2)	C9A—H9A	0.9500
C4—H4	0.9500	C9A—C10A	1.363 (15)
C4—C5	1.397 (2)	C10A—H10A	0.9500
C5—H5A	0.9500	C10A—C11A	1.382 (13)
C5—C6	1.382 (2)	C11A—H11A	0.9500
C7—C8	1.382 (3)	C11A—C12A	1.370 (13)
C7—C12	1.409 (2)	C13A—C14A	1.369 (14)
C8—H8	0.9500	C13A—C18A	1.382 (13)
C8—C9	1.399 (3)	C14A—H14A	0.9500
C9—H9	0.9500	C14A—C15A	1.375 (15)
C9—C10	1.396 (2)	C15A—H15A	0.9500
C10—H10	0.9500	C15A—C16A	1.378 (15)
C10—C11	1.399 (2)	C16A—H16A	0.9500
C11—H11	0.9500	C16A—C17A	1.387 (12)
C11—C12	1.382 (2)	C17A—H17A	0.9500
C13—C14	1.392 (3)	C17A—C18A	1.365 (13)
			
H19A—C19—H19B	109.5	C16—C17—H17	120.9
H19A—C19—H19C	109.5	C18—C17—C16	118.20 (14)
H19B—C19—H19C	109.5	C18—C17—H17	120.9
C20—C19—H19A	109.5	N6—C18—C13	108.64 (17)
C20—C19—H19B	109.5	C17—C18—N6	130.44 (16)
C20—C19—H19C	109.5	C17—C18—C13	120.92 (13)
O1—C20—C19	121.34 (13)	N1—B1—N2	107.25 (15)
O1—C20—C21	121.53 (13)	N1—B1—N6	133.95 (16)
C21—C20—C19	117.12 (12)	N6—B1—N2	118.79 (13)
C20—C21—H21A	109.5	N2—B2—N4	118.80 (13)
C20—C21—H21B	109.5	N3—B2—N2	133.35 (16)
C20—C21—H21C	109.5	N3—B2—N4	107.85 (15)
H21A—C21—H21B	109.5	N4—B3—N6	118.71 (12)
H21A—C21—H21C	109.5	N5—B3—N4	133.60 (16)
H21B—C21—H21C	109.5	N5—B3—N6	107.68 (14)
H22A—C22—H22B	109.5	C1A—N1A—H1A	122 (8)
H22A—C22—H22C	109.5	C1A—N1A—B1A	105.0 (9)
H22B—C22—H22C	109.5	B1A—N1A—H1A	133 (8)
C23—C22—H22A	109.5	B1A—N2A—C6A	107.3 (9)
C23—C22—H22B	109.5	B1A—N2A—B2A	121.9 (9)
C23—C22—H22C	109.5	B2A—N2A—C6A	130.8 (9)
O2—C23—C22	122.38 (13)	C7A—N3A—H3AA	126.2
O2—C23—C24	120.99 (13)	C7A—N3A—B2A	107.6 (10)
C22—C23—C24	116.61 (13)	B2A—N3A—H3AA	126.2
C23—C24—H24A	109.5	B2A—N4A—C12A	106.9 (9)
C23—C24—H24B	109.5	B3A—N4A—C12A	130.8 (10)
C23—C24—H24C	109.5	B3A—N4A—B2A	122.2 (8)
H24A—C24—H24B	109.5	C13A—N5A—H5AA	106 (8)
H24A—C24—H24C	109.5	C13A—N5A—B3A	107.1 (9)
H24B—C24—H24C	109.5	B3A—N5A—H5AA	147 (8)
C1—N1—H1	121.2 (10)	B1A—N6A—C18A	130.6 (10)
C1—N1—B1	107.84 (14)	B1A—N6A—B3A	122.9 (9)
B1—N1—H1	130.4 (10)	B3A—N6A—C18A	106.5 (9)
C6—N2—B1	106.69 (14)	C2A—C1A—N1A	123.9 (12)
C6—N2—B2	132.10 (15)	C6A—C1A—N1A	113.6 (10)
B2—N2—B1	121.21 (13)	C6A—C1A—C2A	122.5 (10)
C7—N3—H3	121.0 (10)	C1A—C2A—H2A	121.3
C7—N3—B2	107.62 (14)	C1A—C2A—C3A	117.3 (12)
B2—N3—H3	131.4 (10)	C3A—C2A—H2A	121.3
C12—N4—B2	106.30 (14)	C2A—C3A—H3AB	120.2
C12—N4—B3	132.59 (15)	C4A—C3A—C2A	119.7 (13)
B3—N4—B2	121.07 (12)	C4A—C3A—H3AB	120.2
C13—N5—H5	120.5 (10)	C3A—C4A—H4A	118.6
C13—N5—B3	107.76 (14)	C3A—C4A—C5A	122.8 (14)
B3—N5—H5	131.7 (10)	C5A—C4A—H4A	118.6
C18—N6—B1	132.24 (15)	C4A—C5A—H5AB	121.4
C18—N6—B3	106.46 (14)	C6A—C5A—C4A	117.2 (13)
B1—N6—B3	121.25 (12)	C6A—C5A—H5AB	121.4
N1—C1—C6	109.50 (16)	C1A—C6A—N2A	106.6 (10)
C2—C1—N1	129.53 (19)	C5A—C6A—N2A	132.9 (12)
C2—C1—C6	120.97 (15)	C5A—C6A—C1A	120.4 (10)
C1—C2—H2	121.1	C8A—C7A—N3A	126.1 (13)
C1—C2—C3	117.88 (17)	C8A—C7A—C12A	122.4 (10)
C3—C2—H2	121.1	C12A—C7A—N3A	111.4 (11)
C2—C3—H3A	119.6	C7A—C8A—H8A	120.7
C4—C3—C2	120.88 (17)	C7A—C8A—C9A	118.5 (12)
C4—C3—H3A	119.6	C9A—C8A—H8A	120.7
C3—C4—H4	119.5	C8A—C9A—H9A	120.9
C3—C4—C5	120.98 (16)	C10A—C9A—C8A	118.3 (15)
C5—C4—H4	119.5	C10A—C9A—H9A	120.9
C4—C5—H5A	120.9	C9A—C10A—H10A	117.7
C6—C5—C4	118.26 (14)	C9A—C10A—C11A	124.6 (16)
C6—C5—H5A	120.9	C11A—C10A—H10A	117.7
C1—C6—N2	108.71 (16)	C10A—C11A—H11A	122.5
C5—C6—N2	130.27 (16)	C12A—C11A—C10A	115.0 (13)
C5—C6—C1	121.01 (13)	C12A—C11A—H11A	122.5
N3—C7—C12	109.33 (17)	C7A—C12A—N4A	106.8 (11)
C8—C7—N3	129.57 (19)	C7A—C12A—C11A	121.1 (10)
C8—C7—C12	121.10 (15)	C11A—C12A—N4A	132.1 (12)
C7—C8—H8	120.9	N5A—C13A—C18A	112.6 (11)
C7—C8—C9	118.21 (18)	C14A—C13A—N5A	126.2 (13)
C9—C8—H8	120.9	C14A—C13A—C18A	121.1 (9)
C8—C9—H9	119.7	C13A—C14A—H14A	122.4
C10—C9—C8	120.5 (2)	C13A—C14A—C15A	115.3 (12)
C10—C9—H9	119.7	C15A—C14A—H14A	122.4
C9—C10—H10	119.4	C14A—C15A—H15A	117.9
C9—C10—C11	121.24 (18)	C14A—C15A—C16A	124.2 (16)
C11—C10—H10	119.4	C16A—C15A—H15A	117.9
C10—C11—H11	121.0	C15A—C16A—H16A	120.1
C12—C11—C10	118.00 (15)	C15A—C16A—C17A	119.9 (15)
C12—C11—H11	121.0	C17A—C16A—H16A	120.1
C7—C12—N4	108.88 (17)	C16A—C17A—H17A	122.0
C11—C12—N4	130.22 (16)	C18A—C17A—C16A	116.0 (12)
C11—C12—C7	120.88 (13)	C18A—C17A—H17A	122.0
N5—C13—C18	109.44 (17)	C13A—C18A—N6A	105.6 (10)
C14—C13—N5	129.36 (19)	C17A—C18A—N6A	130.9 (12)
C14—C13—C18	121.20 (15)	C17A—C18A—C13A	123.5 (9)
C13—C14—H14	121.2	N2A—B1A—N1A	107.4 (9)
C13—C14—C15	117.67 (18)	N6A—B1A—N1A	134.6 (11)
C15—C14—H14	121.2	N6A—B1A—N2A	118.0 (9)
C14—C15—H15	119.5	N2A—B2A—N4A	117.6 (8)
C14—C15—C16	121.0 (2)	N3A—B2A—N2A	135.0 (11)
C16—C15—H15	119.5	N3A—B2A—N4A	107.2 (9)
C15—C16—H16	119.5	N4A—B3A—N6A	117.2 (8)
C17—C16—C15	121.01 (17)	N5A—B3A—N4A	134.7 (10)
C17—C16—H16	119.5	N5A—B3A—N6A	108.1 (9)
			
N1—C1—C2—C3	−178.74 (15)	N1A—C1A—C2A—C3A	−178.3 (14)
N1—C1—C6—N2	−0.09 (15)	N1A—C1A—C6A—N2A	−0.7 (14)
N1—C1—C6—C5	178.83 (11)	N1A—C1A—C6A—C5A	177.6 (11)
N3—C7—C8—C9	178.04 (17)	N3A—C7A—C8A—C9A	179.9 (15)
N3—C7—C12—N4	1.36 (14)	N3A—C7A—C12A—N4A	−0.1 (13)
N3—C7—C12—C11	−177.48 (11)	N3A—C7A—C12A—C11A	−179.9 (11)
N5—C13—C14—C15	178.95 (17)	N5A—C13A—C14A—C15A	177.8 (15)
N5—C13—C18—N6	0.09 (14)	N5A—C13A—C18A—N6A	1.8 (12)
N5—C13—C18—C17	−179.75 (11)	N5A—C13A—C18A—C17A	−178.2 (10)
C1—N1—B1—N2	0.79 (14)	C1A—N1A—B1A—N2A	0.4 (12)
C1—N1—B1—N6	−179.44 (14)	C1A—N1A—B1A—N6A	−177.3 (13)
C1—C2—C3—C4	−0.9 (2)	C1A—C2A—C3A—C4A	−2 (3)
C2—C1—C6—N2	179.59 (13)	C2A—C1A—C6A—N2A	178.1 (12)
C2—C1—C6—C5	−1.5 (2)	C2A—C1A—C6A—C5A	−4 (2)
C2—C3—C4—C5	0.1 (2)	C2A—C3A—C4A—C5A	1 (3)
C3—C4—C5—C6	0.2 (2)	C3A—C4A—C5A—C6A	−1 (2)
C4—C5—C6—N2	179.21 (13)	C4A—C5A—C6A—N2A	−179.6 (13)
C4—C5—C6—C1	0.55 (19)	C4A—C5A—C6A—C1A	3 (2)
C6—N2—B1—N1	−0.83 (14)	C6A—N2A—B1A—N1A	−0.9 (12)
C6—N2—B1—N6	179.35 (11)	C6A—N2A—B1A—N6A	177.3 (9)
C6—N2—B2—N3	−2.1 (3)	C6A—N2A—B2A—N3A	−2 (2)
C6—N2—B2—N4	176.60 (12)	C6A—N2A—B2A—N4A	−177.4 (10)
C6—C1—C2—C3	1.7 (2)	C6A—C1A—C2A—C3A	3 (2)
C7—N3—B2—N2	178.35 (14)	C7A—N3A—B2A—N2A	−177.2 (12)
C7—N3—B2—N4	−0.47 (14)	C7A—N3A—B2A—N4A	−1.9 (12)
C7—C8—C9—C10	0.1 (3)	C7A—C8A—C9A—C10A	2 (3)
C8—C7—C12—N4	−178.79 (13)	C8A—C7A—C12A—N4A	−179.0 (12)
C8—C7—C12—C11	2.4 (2)	C8A—C7A—C12A—C11A	1 (2)
C8—C9—C10—C11	1.1 (3)	C8A—C9A—C10A—C11A	−2 (3)
C9—C10—C11—C12	−0.5 (2)	C9A—C10A—C11A—C12A	2 (3)
C10—C11—C12—N4	−179.74 (14)	C10A—C11A—C12A—N4A	178.8 (15)
C10—C11—C12—C7	−1.2 (2)	C10A—C11A—C12A—C7A	−2 (2)
C12—N4—B2—N2	−177.75 (11)	C12A—N4A—B2A—N2A	178.1 (9)
C12—N4—B2—N3	1.27 (14)	C12A—N4A—B2A—N3A	1.9 (11)
C12—N4—B3—N5	−0.4 (2)	C12A—N4A—B3A—N5A	−3 (2)
C12—N4—B3—N6	−179.55 (12)	C12A—N4A—B3A—N6A	178.5 (9)
C12—C7—C8—C9	−1.8 (2)	C12A—C7A—C8A—C9A	−1 (2)
C13—N5—B3—N4	179.53 (14)	C13A—N5A—B3A—N4A	179.4 (12)
C13—N5—B3—N6	−1.21 (14)	C13A—N5A—B3A—N6A	−1.8 (12)
C13—C14—C15—C16	0.6 (3)	C13A—C14A—C15A—C16A	1 (3)
C14—C13—C18—N6	179.70 (14)	C14A—C13A—C18A—N6A	−179.7 (12)
C14—C13—C18—C17	−0.1 (2)	C14A—C13A—C18A—C17A	0 (2)
C14—C15—C16—C17	0.0 (3)	C14A—C15A—C16A—C17A	−1 (3)
C15—C16—C17—C18	−0.7 (2)	C15A—C16A—C17A—C18A	0 (2)
C16—C17—C18—N6	−179.02 (13)	C16A—C17A—C18A—N6A	179.9 (13)
C16—C17—C18—C13	0.78 (19)	C16A—C17A—C18A—C13A	−0.1 (19)
C18—N6—B1—N1	0.6 (2)	C18A—N6A—B1A—N1A	0 (2)
C18—N6—B1—N2	−179.62 (12)	C18A—N6A—B1A—N2A	−177.5 (9)
C18—N6—B3—N4	−179.36 (11)	C18A—N6A—B3A—N4A	−178.1 (9)
C18—N6—B3—N5	1.26 (13)	C18A—N6A—B3A—N5A	2.8 (11)
C18—C13—C14—C15	−0.6 (3)	C18A—C13A—C14A—C15A	−1 (2)
B1—N1—C1—C2	179.91 (15)	B1A—N1A—C1A—C2A	−178.6 (13)
B1—N1—C1—C6	−0.45 (14)	B1A—N1A—C1A—C6A	0.2 (13)
B1—N2—C6—C1	0.57 (14)	B1A—N2A—C6A—C1A	1.0 (13)
B1—N2—C6—C5	−178.21 (13)	B1A—N2A—C6A—C5A	−177.0 (14)
B1—N2—B2—N3	178.83 (14)	B1A—N2A—B2A—N3A	178.8 (12)
B1—N2—B2—N4	−2.45 (18)	B1A—N2A—B2A—N4A	3.8 (16)
B1—N6—C18—C13	−178.00 (13)	B1A—N6A—C18A—C13A	174.6 (11)
B1—N6—C18—C17	1.8 (2)	B1A—N6A—C18A—C17A	−5.4 (19)
B1—N6—B3—N4	−1.81 (18)	B1A—N6A—B3A—N4A	4.2 (16)
B1—N6—B3—N5	178.81 (11)	B1A—N6A—B3A—N5A	−174.8 (9)
B2—N2—C6—C1	−178.58 (13)	B2A—N2A—C6A—C1A	−178.0 (11)
B2—N2—C6—C5	2.6 (2)	B2A—N2A—C6A—C5A	4 (2)
B2—N2—B1—N1	178.43 (11)	B2A—N2A—B1A—N1A	178.2 (9)
B2—N2—B1—N6	−1.38 (18)	B2A—N2A—B1A—N6A	−3.7 (16)
B2—N3—C7—C8	179.63 (15)	B2A—N3A—C7A—C8A	−179.9 (13)
B2—N3—C7—C12	−0.53 (14)	B2A—N3A—C7A—C12A	1.3 (13)
B2—N4—C12—C7	−1.60 (13)	B2A—N4A—C12A—C7A	−1.1 (12)
B2—N4—C12—C11	177.09 (13)	B2A—N4A—C12A—C11A	178.6 (13)
B2—N4—B3—N5	177.03 (13)	B2A—N4A—B3A—N5A	174.7 (11)
B2—N4—B3—N6	−2.16 (18)	B2A—N4A—B3A—N6A	−4.0 (15)
B3—N4—C12—C7	176.07 (12)	B3A—N4A—C12A—C7A	176.6 (11)
B3—N4—C12—C11	−5.2 (2)	B3A—N4A—C12A—C11A	−4 (2)
B3—N4—B2—N2	4.25 (18)	B3A—N4A—B2A—N2A	0.2 (15)
B3—N4—B2—N3	−176.73 (11)	B3A—N4A—B2A—N3A	−176.1 (9)
B3—N5—C13—C14	−178.86 (16)	B3A—N5A—C13A—C14A	−178.4 (13)
B3—N5—C13—C18	0.71 (14)	B3A—N5A—C13A—C18A	0.0 (13)
B3—N6—C18—C13	−0.83 (14)	B3A—N6A—C18A—C13A	−2.8 (11)
B3—N6—C18—C17	178.99 (13)	B3A—N6A—C18A—C17A	177.2 (12)
B3—N6—B1—N1	−176.20 (13)	B3A—N6A—B1A—N1A	177.0 (11)
B3—N6—B1—N2	3.55 (18)	B3A—N6A—B1A—N2A	−0.5 (16)

### Hydrogen-bond geometry (Å, º)

**Table:**

*D*—H···*A*	*D*—H	H···*A*	*D*···*A*	*D*—H···*A*
N3—H3···O2	0.89 (1)	2.13 (1)	2.9817 (14)	160 (1)
N5—H5···O1	0.87 (1)	2.09 (1)	2.9544 (15)	175 (1)
N1*A*—H1*A*···O2	0.88 (2)	2.27 (4)	3.133 (8)	165 (11)
N5*A*—H5*AA*···O1	0.88 (2)	2.19 (5)	3.026 (8)	158 (11)
